# Supplementary material for: The Anti-Virulence Effect of Vismia guianensis against Candida albicans and Candida glabrata
Source: Antibiotics (Basel). 2022 Dec 16;11(12):1834. doi: 10.3390/antibiotics11121834 (PMC9774440; doi:10.3390/antibiotics11121834)
Supplement: Supplementary file 1 [file antibiotics-11-01834-s001.zip › antibiotics-2058226-Supplementary result.pdf]

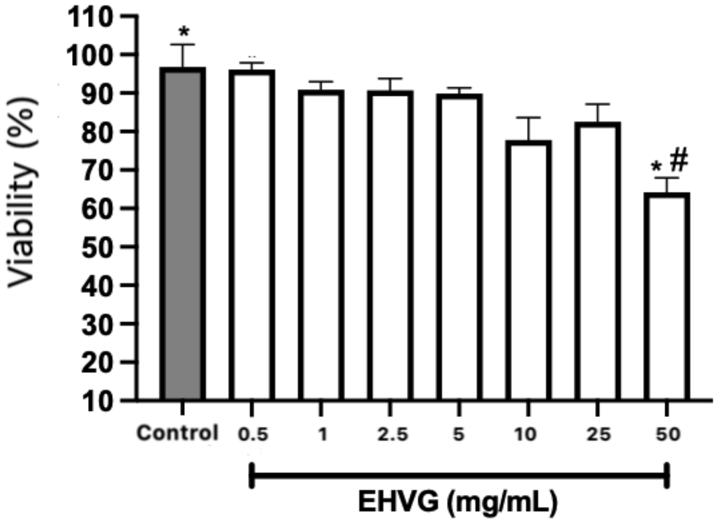

Figure S1. Effect of different concentrations of the hydroalcoholic leaf extract of *Vismia guianensis* (EHVG) on RAW 264.7 cells viability tested by neutral red assay. Medium was used as negative control. Data represent the mean ± standard deviation of individual samples tested in quadruplicate. (*) $p<0.05$ compared to the control; (#) $p<0.05$ compared to EHVG 0.5mg/mL.
